# Advances in Molecular Mechanisms and Therapeutic Strategies in Colorectal Cancer: A New Era of Precision Medicine

**DOI:** 10.3390/ijms26010346

**Published:** 2025-01-02

**Authors:** Donatella Delle Cave

**Affiliations:** Institute of Genetics and Biophysics ‘Adriano Buzzati-Traverso’, National Research Council CNR, 80131 Naples, Italy; donatella.dellecave@igb.cnr.it

## 1. Introduction

Colorectal cancer (CRC) is one of the leading causes of cancer-related morbidity and mortality worldwide [1]. Despite significant advancements in early detection, surgical techniques, and systemic therapies, CRC continues to present major clinical challenges due to its heterogeneous nature and the complexity of its molecular mechanisms [2]. The survival rates for CRC have improved over the past few decades, yet the disease remains a major health challenge. Part of the reason for the continued high mortality is the heterogeneity of the disease, which encompasses a wide range of molecular subtypes, varying genetic mutations, and distinct tumor microenvironments [3,4]. However, recent breakthroughs in molecular biology and precision medicine are beginning to transform the landscape of colorectal cancer treatment, offering new hope for more effective and personalized therapies [5].

## 2. Molecular Drivers and Prognostic Markers

CRC is characterized by a high degree of genetic and epigenetic variability, with molecular alterations influencing every stage of tumorigenesis, from initiation to metastasis [6]. The genetic landscape of CRC is dominated by mutations in key oncogenes and tumor suppressor genes, along with alterations in signaling pathways that regulate cell proliferation, survival, differentiation, and apoptosis. Mutations in the adenomatous polyposis coli (APC) gene are among the earliest genetic events in CRC, leading to the activation of the Wnt/β-catenin signaling pathway, which drives the development of adenomatous polyps and eventually carcinoma [7]. Additionally, mutations in KRAS, TP53, and PIK3CA contribute to the progression of CRC, while the SMAD4 and TGF-β signaling pathways are frequently altered in metastatic disease [8,9]. The PI3K/Akt pathway is often dysregulated in CRC, with mutations in the *PIK3CA* gene leading to enhanced cell survival and proliferation. Świechowski R. and colleagues, examined the expression of key genes in this pathway (*PIK3CA*, *PTEN*, *AKT1*, and *FOXO1*) in CRC patients and found that *FOXO1* expression was higher in older patients and rectal tumors [10]. A loss of *PTEN* function was linked to higher malignancy and neuroinvasion. A bioinformatic analysis showed that *PTEN* and *FOXO1* were more highly expressed in normal tissue than tumor tissue, suggesting a tumor-suppressive role. The study also explored molecular subtypes of CRC, finding that the mesenchymal subtype had the highest PIK3CA expression, associated with poor prognosis. Higher PTEN expression was linked to better relapse-free survival (RFS), but surprisingly, a worse overall survival (OS). The findings suggest that alterations in the PI3K/Akt pathway could serve as prognostic markers, guiding personalized treatments. Tsumuraya H. and colleagues used an in silico approach to analyze multiple transcriptomic datasets of colorectal precursor lesions and CRCs, aiming to investigate TGFβ-induced stromal activation in the serrated pathway [11]. A multi-gene signature (TBSS) was employed to assess TGFβ-induced stromal activation, with validation confirming its specificity to the tumor stroma, excluding epithelial signals. The authors showed that serrated CRCs exhibited significantly higher TBSS levels compared to conventional CRCs, with BRAF-mutant CRCs showing elevated TBSS levels compared to BRAFwild-type CRCs. The microsatellite instability (MSI) pathway, associated with defects in the DNA mismatch repair system, is a hallmark of certain CRC subtypes, particularly in hereditary non-polyposis colorectal cancer (HNPCC), also known as Lynch syndrome [12]. MSI is characterized by the accumulation of mutations in short, repetitive DNA sequences, which leads to tumor heterogeneity and can influence both prognosis and therapeutic responses. In addition to genetic mutations, epigenetic changes, such as DNA methylation and histone modifications, play a critical role in CRC [13]. The aberrant promoter methylation of tumor suppressor genes, such as MLH1 and MGMT, can silence their expression, contributing to tumorigenesis [14]. Furthermore, the altered expression of non-coding RNAs, including microRNAs and long non-coding RNAs, has been implicated in CRC development, providing new avenues for biomarker discovery and therapeutic targeting [15]. The tumor microenvironment also remains a largely untapped area of exploration.

## 3. Microenvironment and Microbiome

CRC tumors are known to have a complex microenvironment characterized by immune cells, fibroblasts, endothelial cells, and extracellular matrix components, which plays a crucial role in CRC progression [16]. Understanding how to manipulate this microenvironment to enhance treatment response is a key area for future research. Additionally, the role of the gut microbiome in CRC progression and treatment response is an emerging field that requires further investigation [17]. Recent studies suggest that the microbiome may influence not only cancer development but also the efficacy of certain therapies, including immunotherapy. Tumor-associated macrophages (TAMs) and myeloid-derived suppressor cells (MDSCs) are known to promote CRC metastasis by enhancing angiogenesis and suppressing antitumor immunity. Additionally, the microbiome has emerged as a key factor in CRC development, with certain gut bacteria, such as Fusobacterium nucleatum, linked to poor prognosis and therapeutic resistance. These discoveries underscore the importance of the TME in influencing CRC metastasis, chemoresistance, and immune evasion [18]. Traditional treatments for CRC, such as surgical resection, chemotherapy, and radiotherapy, remain the cornerstone of care for many patients. However, the high recurrence rate and development of resistance to chemotherapy have prompted the search for more effective, targeted therapies [19].

## 4. Chemotherapy and Targeted Therapy

Chemotherapy regimens, such as FOLFOX (5-fluorouracil, leucovorin, and oxaliplatin) and FOLFIRI (5-fluorouracil, leucovorin, and irinotecan), are widely used in the treatment of advanced CRC, particularly in metastatic settings [20]. Although these regimens can provide survival benefits, they often come with significant side effects, and their efficacy is limited in some patients due to intrinsic or acquired resistance mechanisms. The FOLFOXIRI chemotherapy regimen, a combination of oxaliplatin (OX), irinotecan (SN-38), and 5-FU, has shown superior survival outcomes but is still limited in effectiveness for advanced CRC [21]. Maxim Girod et al. explored the combination of FOLFOXIRI and the oncolytic virus CVB3 PD-H, finding that PD-H synergizes with the chemotherapy drugs, enhancing cytotoxicity in the CRC Colo320 cell line [22]. Timing is crucial in combination therapies, and the authors suggest administering PD-H 24-48 h before FOLFOXIRI to maximize synergy. This combination may also facilitatea reduction in chemotherapy doses, potentially minimizing side effects, especially for patients who cannot tolerate the full regimen. Further research is needed to confirm these findings in vivo, but the combination of FOLFOXIRI and PD-H shows promise not only in enhancing tumor killing but also in triggering an immune response against CRC and metastases. The discovery of specific molecular alterations in CRC has led to the development of targeted therapies that aim to inhibit key signaling pathways involved in tumor growth. Epidermal growth factor receptor (EGFR) inhibitors, such as cetuximab and panitumumab, have shown efficacy in patients with KRAS wild-type tumors [23]. Similarly, vascular endothelial growth factor (VEGF) inhibitors, such as bevacizumab, target the angiogenesis process, limiting the tumor blood supply and reducing metastasis [24]. However, resistance to targeted therapies remains a major challenge, particularly in tumors harboring mutations in KRAS, NRAS, and BRAF [25]. These mutations can activate downstream signaling pathways, rendering EGFR inhibitors ineffective. As a result, research is currently focused on developing second- and third-generation inhibitors, such as BRAF inhibitors and MEK inhibitors, for patients with BRAF-mutant CRC. Immunotherapy has emerged as a promising treatment option for certain CRC patients, particularly those with MSI-high (MSI-H) tumors [26]. Checkpoint inhibitors such as pembrolizumab and nivolumab, which target the PD-1/PD-L1 pathway, have shown significant clinical benefit in MSI-H CRC, leading to their approval for the treatment of these patients [27]. However, only a subset of CRC patients benefit from immunotherapy, and strategies to overcome resistance are actively being explored, including combination approaches with other immune modulators, targeted therapies, and chemotherapy.

## 5. Liquid Biopsy

The development of liquid biopsy techniques, which detect tumor-derived genetic material in blood or other bodily fluids, holds great promise for the early detection and monitoring of CRC. Liquid biopsy can be used to detect circulating tumor DNA (ctDNA) or circulating tumor cells (CTCs), facilitating non-invasive screening, the detection of minimal residual disease, and the assessment of treatment response [28]. Moreover, liquid biopsy has the potential to identify mutations associated with drug resistance, providing real-time insight into tumor evolution and facilitating more personalized treatment strategies. The future of CRC treatment lies in the development of personalized medicine, where therapies are tailored to the molecular profile of individual tumors. This approach involves the use of genomic and molecular testing to identify actionable mutations, predict treatment response, and monitor disease progression. Emerging technologies, such as next-generation sequencing (NGS), single-cell RNA sequencing, and CRISPR-based gene editing, are expected to accelerate our understanding of CRC at a molecular level, enabling the identification of new therapeutic targets and biomarkers [29,30]. In addition, combination therapies that target multiple pathways simultaneously, including the use of targeted therapies, immunotherapies, and chemotherapy, are showing promise in overcoming resistance and improving patient outcomes.

## 6. Nanoparticle-Based Therapies and Precision Medicine

Ongoing clinical trials and research into novel drug delivery systems, such as nanoparticle-based therapies, are also opening new avenues for enhancing treatment efficacy while minimizing side effects [31,32,33]. Recently, Delle Cave et al. developed a hybrid nanoplatform for targeted therapy against metastatic CRC [31]. The nanoplatform combines gelatin-embedded drug-loaded nanoparticles (DNPs), gold nanoparticles (AuNPs), and the anticancer drug Galunisertib (LY). It is functionalized with antibodies targeting L1CAM, a marker found in metastasis-initiating cells, ensuring the precise targeting of cancer cells and reducing off-target effects. The platform’s effectiveness was tested in vitro and in vivo. In cell cultures, the nanoplatform selectively targeted L1CAM-positive CRC cells, effectively delivering the drug and reducing mesenchymal traits and invasiveness. In vivo, intratumoral injection significantly reduced tumor growth, with a histological analysis showing the inhibition of the mesenchymal phenotype and necrotic tissue in treated tumors. Raman imaging revealed a better distribution and higher accumulation of the targeted nanoplatform in tumors compared to untargeted nanoparticles. In conclusion, the landscape of colorectal cancer treatment is undergoing a transformative shift with the advent of precision medicine. Molecular insights into the genetic drivers of CRC, along with advances in targeted therapies, immunotherapies, and combination treatments, are providing new hope for more effective and personalized interventions. However, several critical gaps remain in our understanding of the disease, particularly with regard to treatment resistance, the tumor microenvironment, and the development of predictive biomarkers. By addressing these gaps through continued research, we can pave the way for a new era in CRC treatment, one in which therapies are tailored to the individual patient, offering better outcomes and improved survival rates.

## References

[1] Xi Y., Xu P. (2021). Global Colorectal Cancer Burden in 2020 and Projections to 2040. Transl. Oncol..

[2] Yang Z., Wang X., Zhou H., Jiang M., Wang J., Sui B. (2024). Molecular Complexity of Colorectal Cancer: Pathways, Biomarkers, and Therapeutic Strategies. Cancer Manag. Res..

[3] Gustavsson B., Carlsson G., Machover D., Petrelli N., Roth A., Schmoll H.-J., Tveit K.-M., Gibson F. (2015). A Review of the Evolution of Systemic Chemotherapy in the Management of Colorectal Cancer. Clin. Color. Cancer.

[4] Rawla P., Sunkara T., Barsouk A. (2019). Epidemiology of Colorectal Cancer: Incidence, Mortality, Survival, and Risk Factors. Gastroenterol. Rev./Przegląd Gastroenterol..

[5] Xie Y.-H., Chen Y.-X., Fang J.-Y. (2020). Comprehensive Review of Targeted Therapy for Colorectal Cancer. Signal Transduct. Target. Ther..

[6] Papavassiliou A.G., Delle Cave D. (2024). Novel Therapeutic Approaches for Colorectal Cancer Treatment. Int. J. Mol. Sci..

[7] Li B., Zhang G., Xu X. (2023). APC Mutation Correlated with Poor Response of Immunotherapy in Colon Cancer. BMC Gastroenterol..

[8] Papavassiliou K.A., Delle Cave D., Papavassiliou A.G. (2023). Targeting the TGF-β Signaling Axis in Metastatic Colorectal Cancer: Where Do We Stand?. Int. J. Mol. Sci..

[9] Cave D.D., Hernando-Momblona X., Sevillano M., Minchiotti G., Lonardo E. (2021). Nodal-Induced L1CAM/CXCR4 Subpopulation Sustains Tumor Growth and Metastasis in Colorectal Cancer Derived Organoids. Theranostics.

[10] Świechowski R., Pietrzak J., Wosiak A., Mik M., Balcerczak E. (2024). Genetic Insights into Colorectal Cancer: Evaluating PI3K/AKT Signaling Pathway Genes Expression. Int. J. Mol. Sci..

[11] Tsumuraya H., Okayama H., Katagata M., Matsuishi A., Fukai S., Ito M., Sakamoto W., Saito M., Momma T., Nakajima S. (2024). TGFβ-Responsive Stromal Activation Occurs Early in Serrated Colorectal Carcinogenesis. Int. J. Mol. Sci..

[12] Vilar E., Gruber S.B. (2010). Microsatellite Instability in Colorectal Cancer—The Stable Evidence. Nat. Rev. Clin. Oncol..

[13] Lao V.V., Grady W.M. (2011). Epigenetics and Colorectal Cancer. Nat. Rev. Gastroenterol. Hepatol..

[14] Zhao N., Lai C., Wang Y., Dai S., Gu H. (2024). Understanding the Role of DNA Methylation in Colorectal Cancer: Mechanisms, Detection, and Clinical Significance. Biochim. Biophys. Acta Rev. Cancer.

[15] Wu Y., Xu X. (2023). Long Non-Coding RNA Signature in Colorectal Cancer: Research Progression and Clinical Application. Cancer Cell Int..

[16] Li J., Chen D., Shen M. (2022). Tumor Microenvironment Shapes Colorectal Cancer Progression, Metastasis, and Treatment Responses. Front. Med..

[17] Kim J., Lee H.K. (2022). Potential Role of the Gut Microbiome in Colorectal Cancer Progression. Front. Immunol..

[18] Zhou D., Li Y. (2023). Gut Microbiota and Tumor-Associated Macrophages: Potential in Tumor Diagnosis and Treatment. Gut Microbes.

[19] McQuade R.M., Stojanovska V., Bornstein J.C., Nurgali K. (2017). Colorectal Cancer Chemotherapy: The Evolution of Treatment and New Approaches. Curr. Med. Chem..

[20] Jonker D., Rumble R.B., Maroun J., the Gastrointestinal Cancer Disease Site Group of Cancer Care Ontario’s Program in Evidence-Based Care (2006). Role of Oxaliplatin Combined with 5-Fluorouracil and Folinic Acid in the First- and Second-Line Treatment of Advanced Colorectal Cancer. Curr. Oncol..

[21] Cervantes A., Prager G.W. (2023). FOLFOXIRI plus Bevacizumab as Standard of Care for First-Line Treatment in Patients with Advanced Colon Cancer. ESMO Open.

[22] Girod M., Geisler A., Hinze L., Elsner L., Dieringer B., Beling A., Kurreck J., Fechner H. (2024). Combination of FOLFOXIRI Drugs with Oncolytic Coxsackie B3 Virus PD-H Synergistically Induces Oncolysis in the Refractory Colorectal Cancer Cell Line Colo320. Int. J. Mol. Sci..

[23] Harada K., Yuki S., Kawamoto Y., Nakamura T., Kaneko S., Ishida K., Sakamoto N., Komatsu Y. (2023). Anti-Epidermal Growth Factor Receptor Treatment for Patients with Neo RAS Wild-Type Metastatic Colorectal Cancer: A Case Report of Two Cases. Ther. Adv. Med. Oncol..

[24] Ghalehbandi S., Yuzugulen J., Pranjol M.Z.I., Pourgholami M.H. (2023). The Role of VEGF in Cancer-Induced Angiogenesis and Research Progress of Drugs Targeting VEGF. Eur. J. Pharmacol..

[25] Dazio G., Epistolio S., Frattini M., Saletti P. (2022). Recent and Future Strategies to Overcome Resistance to Targeted Therapies and Immunotherapies in Metastatic Colorectal Cancer. J. Clin. Med..

[26] Ros J., Baraibar I., Saoudi N., Rodriguez M., Salvà F., Tabernero J., Élez E. (2023). Immunotherapy for Colorectal Cancer with High Microsatellite Instability: The Ongoing Search for Biomarkers. Cancers.

[27] Sahin I.H., Akce M., Alese O., Shaib W., Lesinski G.B., El-Rayes B., Wu C. (2019). Immune Checkpoint Inhibitors for the Treatment of MSI-H/MMR-D Colorectal Cancer and a Perspective on Resistance Mechanisms. Br. J. Cancer.

[28] Tan C.R.C., Zhou L., El-Deiry W.S. (2016). Circulating Tumor Cells Versus Circulating Tumor DNA in Colorectal Cancer: Pros and Cons. Curr. Color. Cancer Rep..

[29] Selvakumar S.C., Preethi K.A., Ross K., Tusubira D., Khan M.W.A., Mani P., Rao T.N., Sekar D. (2022). CRISPR/Cas9 and next Generation Sequencing in the Personalized Treatment of Cancer. Mol. Cancer.

[30] Abbes S., Baldi S., Sellami H., Amedei A., Keskes L. (2023). Molecular Methods for Colorectal Cancer Screening: Progress with next-Generation Sequencing Evolution. World J. Gastrointest. Oncol..

[31] Delle Cave D., Mangini M., Tramontano C., De Stefano L., Corona M., Rea I., De Luca A.C., Lonardo E. (2024). Hybrid Biosilica Nanoparticles for In-Vivo Targeted Inhibition of Colorectal Cancer Growth and Label-Free Imaging. Int. J. Nanomed..

[32] Narayana S., Gowda B.H.J., Hani U., Shimu S.S., Paul K., Das A., Ashique S., Ahmed M.G., Tarighat M.A., Abdi G. (2024). Inorganic Nanoparticle-Based Treatment Approaches for Colorectal Cancer: Recent Advancements and Challenges. J. Nanobiotechnology.

[33] Jain A., Bhattacharya S. (2023). Recent Advances in Nanomedicine Preparative Methods and Their Therapeutic Potential for Colorectal Cancer: A Critical Review. Front. Oncol..

